# The segment as the minimal planning unit in speech production and reading aloud: evidence and implications

**DOI:** 10.3389/fpsyg.2015.01457

**Published:** 2015-09-29

**Authors:** Alan H. Kawamoto, Qiang Liu, Christopher T. Kello

**Affiliations:** ^1^Department of Psychology, University of California, Santa CruzSanta Cruz, CA, USA; ^2^Department of Cognitive Science, University of California, MercedMerced, CA, USA

**Keywords:** absolute latency, segment duration, serial vs. parallel encoding

## Abstract

Speech production and reading aloud studies have much in common, especially the last stages involved in producing a response. We focus on the minimal planning unit (MPU) in articulation. Although most researchers now assume that the MPU is the syllable, we argue that it is at least as small as the segment based on negative response latencies (i.e., response initiation before presentation of the complete target) and longer initial segment durations in a reading aloud task where the initial segment is primed. We also discuss why such evidence was not found in earlier studies. Next, we rebut arguments that the segment cannot be the MPU by appealing to flexible planning scope whereby planning units of different sizes can be used due to individual differences, as well as stimulus and experimental design differences. We also discuss why negative response latencies do not arise in some situations and why anticipatory coarticulation does not preclude the segment MPU. Finally, we argue that the segment MPU is also important because it provides an alternative explanation of results implicated in the serial vs. parallel processing debate.

In reading aloud and speech production experiments, participants produce a single word utterance as the response and thus the last processing stages—phonological encoding (assigning a segment to a position in a metrical frame), phonetic encoding (retrieving the motor plans required for articulation), and articulation (producing the gestures leading to an acoustic response)—are shared. Moreover, the 2 fields became closer as speech production researchers began to use chronometric measures (Meyer, 1992) and word reading researchers began to use error measures (Kello and Plaut, 2000). Also, models integrating both fields were being proposed (Roelofs, 2004).

One aspect of processing common to both fields is the degree to which processing is incremental. Incremental processing can be manifested in two non-mutually exclusive ways: (1) a segment (i.e., a consonant or vowel) as the minimal planning unit (MPU) (Kawamoto et al., 1998; Kawamoto, 1999), and (2) cascaded processing (Kello et al., 2000; Rapp and Goldrick, 2000). In this review, we consider the MPU.

Some researchers argue that articulation cannot begin before the currently queued word or syllable is fully planned, while others contend that articulation can start with just one segment planned. We begin by reviewing a variety of phonological units that have been proposed as the MPU, but focus on the segment. Next, we summarize more evidence for the segment and rebut arguments against the segment. Finally, we discuss how the segment MPU provides alternative interpretations of results relevant to the serial vs. parallel processing debate in reading aloud.

## Possible MPUs

Levelt (1989) initially assumed that the MPU was the phonological word—a stress group that may include multiple words. Under this assumption, the phonological word is completely phonologically encoded before it is sent to the phonetic encoding stage. After all syllables have been phonetically encoded, the motor plan for the entire word is sent to the articulator^1^.

However, most researchers now assume that the syllable is the MPU (Schriefers and Teruel, 1999; Meyer et al., 2003). Under this assumption, the initial syllable is phonetically encoded after it has been phonologically encoded and then executed by the articulators after the entire word has been phonologically encoded. For Levelt's (1989; Levelt et al., 1999a) model, the syllable plays a unique role because the motor plan determined at the phonetic encoding stage is based on the syllable (either retrieved from a mental syllabary or assembled from the motor plans of individual segments). Levelt's speech production model has been implemented as the WEAVER model (Levelt et al., 1999a).

Models of reading aloud have also been implemented as computational models (Coltheart et al., 2001; Kello and Plaut, 2003; Perry et al., 2007). Unlike speech production models, however, models of reading aloud focus on the mapping from the spelling to a phonological representation and typically base response latency predictions on the time to generate the phonological representation.

## Segment as the MPU

Various subsyllabic units have been considered as MPUs including the initial consonant(s) or the initial plosive consonant and following vowel (Frederiksen and Kroll, 1976) and the segment, the unit we focus on in this review (MacKay, 1987; Dell et al., 1993; Kawamoto et al., 1998)^2^. If the segment is the MPU, then the motor plan for the initial segment can be retrieved and executed as soon as it has been phonologically encoded. Segment motor plans are part of Levelt's (1989) model, but are not intended to be executed individually.

It is theoretically straightforward to show that the segment is the MPU if phonological or other processes can be shown to affect absolute response latencies and initial segment durations. However, it is methodologically difficult to do so because many initial segments produce little or no acoustic energy.

### Problems due to acoustic characteristics of the initial segment

The biggest problem is that the initial part of plosive and affricate segments are silent. In fact, for plosives, acoustic energy is not generated until the end of the segment when the second segment begins. Because there is no acoustic energy throughout the entire plosive segment, acoustic latency (response latency based on acoustic onset) conflates response latency and initial segment duration. Moreover, matching the initial segment across conditions isn't a solution because any factor that affects initial segment duration affects acoustic latency.

The conflation of response latency and initial segment duration extends to voiceless affricates and fricatives if voice-keys are used because voice-keys typically miss the low intensity acoustic energy of these segments (Pechmann et al., 1989; Sakuma et al., 1997). In fact, the first 2 segments might be missed if the target begins with /s/ followed by a plosive (Sakuma et al., 1997; Rastle and Davis, 2002).

The problems with using acoustic energy to assess processing difficulty arise because the onset of acoustic energy is arguably the last event occurring during articulation. Two alternatives are to index response latency to the initiation of muscular activity using electromyography (Riès et al., 2012) or movement of speech articulators (lips and jaw) using video (Kawamoto et al., 2008). The latter was used in the experiment described below.

### Negative response latencies

To demonstrate that the segment is the MPU, the initial segment of a monosyllabic target word was primed in a reading aloud task (Kawamoto et al., 2014, Expt. 2). The initial letter was followed by underscores and presented for either 300 or 600 ms, at which time the underscores were replaced by the remaining letters of the target. The segment MPU predicts that a response can be initiated before the complete target is presented—resulting in a *negative* response latency—but the syllable MPU does not. The results below are from the 600 ms condition where there is sufficient time for a response to be initiated. Using acoustic onset, 2.5% of the trials had negative latencies measured from onset of the complete target, all beginning with non-plosives. However, using articulatory onset based on movement of the lips and jaw, 26.2% of the trials had negative latencies and these trials included plosives and non-plosives. These negative latencies provide unequivocal evidence for the segment MPU because the initial segment was provided early and measurements were able to detect its initiation early in the course of articulation.

### Initial segment duration differences

Additional evidence for the segment MPU is acoustic durations of responses. Duration effects arise because articulation of the current unit is prolonged while the speaker prepares the following unit to be articulated. Processing effects can be manifested as duration effects in different ways (see Kello, 2004), including duration effects measured across the entire word (Damian, 2003)^3^. However, a duration effect localized to the initial segment is the strongest evidence for the segment as the MPU (Kawamoto et al., 2014).

Although the duration of the initial segment can be measured directly from the acoustic response only for non-plosives, the duration effect for plosives can be determined indirectly. In particular, the *difference* in the duration of initial plosive segments, ISD_P_, in different priming conditions corresponds to the difference in acoustic latency for plosive and non-plosive segments, AL_P_ and AL_N_, respectively:

(1a) $${\text{ISD}}_{\text{P}}\prime \prime -{\text{ISD}}_{\text{P}}\prime =({\text{AL}}_{\text{P}}\prime \prime -{\text{AL}}_{\text{N}}\prime \prime )-({\text{AL}}_{\text{P}}\prime -{\text{AL}}_{\text{N}}\prime ),\text{ and}$$

(1b) $${\text{ISD}}_{\text{P}}\prime \prime -{\text{ISD}}_{\text{P}}\prime =({\text{AL}}_{\text{P}}\prime \prime -{\text{AL}}_{\text{P}}\prime )-({\text{AL}}_{\text{N}}\prime \prime -{\text{AL}}_{\text{N}}\prime ),$$

where double prime and prime denote the 600 ms and 300 ms prime durations, respectively (see also Kawamoto et al., 1998; Kawamoto, 1999).

Alternatively, the initial plosive duration can be determined by using articulatory onset to approximate the beginning of the segment and acoustic onset as the end of the segment. Using both of these approaches, the duration of plosives was also shown to be longer in the 600 ms than in the 300 ms condition due to early initiation of articulation (Kawamoto et al., 2014).

### Rebutting evidence against segment MPU

There are many studies demonstrating that a planning unit larger than the initial segment is used for different stimuli under various experimental conditions. These units include the syllable (Cholin et al., 2011), the initial fragment up to and including the first stressed syllable (Sulpizio et al., 2015), the word (Meyer et al., 2003), two phonological words (Damian and Dumay, 2007), and even the clause (Ferreira and Swets, 2002). These results demonstrate that planning units are variable, and can be as large as the clause.

We argue that the segment remains viable as the MPU because the planning unit varies by individuals, as well as stimuli and experimental design. Two different scenarios can arise with a variable planning scope. In one scenario, an effect can be found assuming a smaller unit than the putative MPU. For example, Damian (2003) found longer word durations when the initial segment was primed as predicted by the segment but not the syllable MPU, but only when a deadline was imposed. In the other scenario, a smaller unit might yield no effect as predicted. For example, monosyllabic words are named as quickly as bisyllabic words when presented in the same block as predicted by syllable and segment MPUs (Meyer et al., 2003; Damian et al., 2010), but more quickly when presented in different blocks as predicted by the word MPU (Meyer et al., 2003). Therefore, the planning unit was ostensibly larger in some studies without finding any effect for the smaller MPU. We further note that a smaller MPU does not always predict shorter latencies; longer latencies can arise if there is competition between different initial syllables (e.g., in assigning stress, Sulpizio et al., 2015) or segments (e.g., in mapping a letter or letters to a phoneme as discussed below).

Another argument is that anticipatory coarticulation precludes the segment MPU because knowledge of upcoming segments is required during articulation and because it is ubiquitous (Levelt et al., 1999b; Rastle et al., 2000). However, Kawamoto and Liu (2007) found that anticipatory coarticulation is not ubiquitous. They had participants utter one member of a minimal pair (still-stool, spill-spool, still-spill, or stool-spool) and found that there was anticipatory coarticulation of the vowel on the initial segment when the vowels were identical, but not when the vowels were different. Moreover, the long interval between articulatory onset and acoustic onset when the initial segment alone is primed (Kawamoto et al., 2014) can be interpreted as coarticulatory effects of the initial segment on the preceding null phoneme.

## Implications of the segment as the MPU

Determining that the segment is the MPU is important in its own right, but it is also important because it provides an alternative account of results in other debates such as whether phonological encoding is purely parallel or has a sequential component. We examine a length effect and a position effect, effects that would be considered straightforward for sequential reading models to account for (e.g., Perry et al., 2007). However, we argue that current sequential models cannot account for the entire pattern of results, but that purely parallel models can if the segment is the MPU and if acoustic characteristics of the initial segment are considered.

### Onset complexity effect

Researchers have examined whether words with a simple onset consisting of a single consonant have shorter or longer naming latencies than words with a complex onset consisting of two or more consonants. An early study by Frederiksen and Kroll (1976) found that when length was controlled, words with simple onsets had shorter naming latencies than words with complex onsets. However, interpreting these results is complicated by two acoustic characteristics of simple and complex onsets. First, complex onsets in English can only begin with plosives or voiceless fricatives, and many complex onsets beginning with /s/ are followed by a plosive. Second, segments have a shorter duration in a complex onset than in a simple onset (Klatt, 1974; Rastle and Davis, 2002).

Kawamoto and Kello (1999) reexamined the onset complexity effect for monosyllabic targets beginning with /s/. (Fillers beginning with plosives were also included.) In one experiment the second consonant of the complex onset was a plosive, and in another it was a non-plosive. Using measures of acoustic latency based on marking digitized responses, they found that targets with complex onsets had *shorter* acoustic latencies than targets with simple onsets despite being *longer* in length. They hypothesized that the inconsistency in their results and Frederiksen and Kroll's (1976) results was due to how acoustic latency was determined. This hypothesis was confirmed by Rastle and Davis (2002) who replicated Kawamoto and Kello's (1999, Expt. 2) results when acoustic latency was based on hand-marking digitized responses, but who found no effect when an integrator voice-key was used, and an opposite effect when a simple voice-key was used (see Table 1).

**Table 1:** **Acoustic latencies of words with simple vs. complex onsets beginning with /s/ followed by a plosive determined in different ways**.

**Kawamoto and Kello (Expt. 2)**						
	**KK digitized**	**RD digitized**	**RD integrator-VK**	**RD simple-VK**	**CDP+**	**DRC**
Simple	462.4	371	449	500	89.8	68
Complex	445.6	362	447	511	94.7	70
Difference	16.8	9	2	−11	−4.9	−2

*Results from Kawamoto and Kello (1999, Expt. 2) (labeled “KK”) based on marking a digitized acoustic waveform, from Rastle and Davis (2002) (labeled “RD”) based on hand-marking a digitized acoustic waveform and 2 different voice-key (VK) methods, and simulation results from the CDP+ model (Perry et al., 2007) and the DRC model (Coltheart et al., 2001)*.

Although the difference in results due to the method of measuring acoustic latency reported by Rastle and Davis has been widely recognized, the theoretical implication of the onset complexity effect has not. In particular, the partially sequential DRC (Coltheart et al., 2001) and CDP+ (Perry et al., 2007) models cannot account for the results (see Table 1) because the sequential rule route of these models processes the input from the beginning to the end of the word 1 letter or 1 grapheme at a time, respectively, independently of other letters and graphemes in the input. Thus, processing takes longer when the input has more letters and graphemes.

However, Kawamoto and Kello argued that the onset complexity result could be accounted for assuming parallel processing if the segment is the MPU. In particular, the initial consonant can be almost any consonant if the 2nd letter is a vowel as it is for simple onsets, but is almost always /s/ if the second segment of a complex onset is a plosive or a nasal consonant. If processing is parallel, the first segment is still being processed when the second segment is being processed. If information about the second segment can influence processing of the first segment, then the initial /s/ of a complex onset followed by a plosive or a nasal consonant should be encoded before the /s/ of a simple onset. Thus, for the segment MPU, articulation can be initiated earlier for targets with complex rather than simple onsets.

### Regularity by position of regularity interaction

Monosyllabic English words with irregular pronunciations have longer acoustic latencies than matched words with regular pronunciations. This regularity effect diminishes as the position of the irregular grapheme moves from left to right (Roberts et al., 2003). The authors argue that sequential models such as the DRC model could account for the data, but purely parallel models could not.

**Figure 1:**
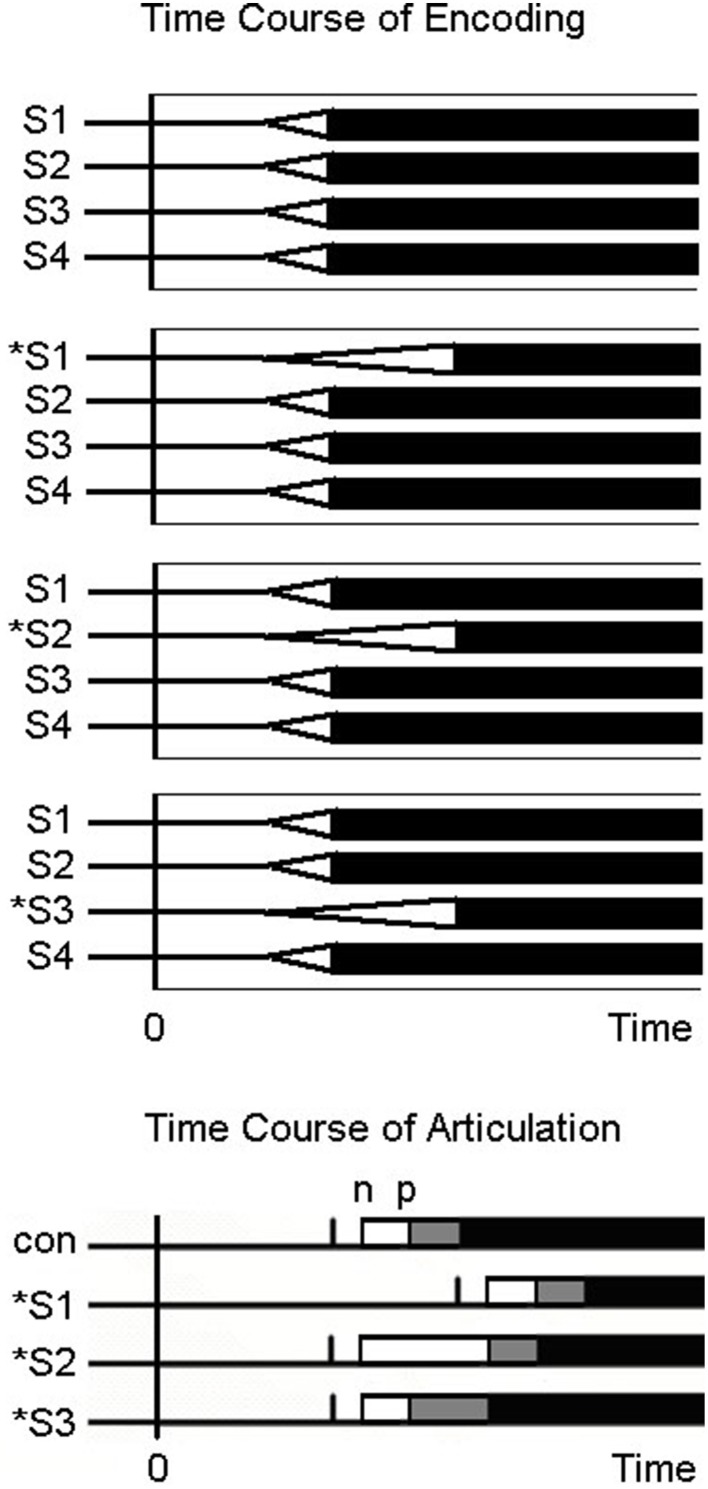
**Time course of encoding and articulation for regular and irregular words**. The top of the figure shows the putative time-course of phonological encoding of monosyllabic words (1 regular word and 3 irregular words) that are 4 segments long (each segment labeled S1, S2, S3, or S4) assuming a parallel encoding scheme. For the 3 irregular words, the irregular segment which occurs at position 1, 2, or 3, is indicated by an “^*^.” On each segment's time-course of phonological encoding, the white triangle depicts the increase in activation of the correct segment, and the base of the triangle on the right side of the triangle depicts when that segment reaches threshold. Below the sets of time-courses of encoding at the bottom of the figure are the time-courses of articulation on the same time-scale as the phonological encoding assuming the segment as the planning unit (i.e., the criterion to initiate articulation). The vertical bar corresponds to the point in time when the 1st segment reaches threshold based on the time-course of encoding above, with the white and gray rectangles corresponding to to the duration of the 1st and 2nd segments. The “n” and “p” at the left and right edges of the white rectangle corresponds to the acoustic onset for non-plosive and plosive initial segments, respectively.

However, all the models considered by Roberts and colleagues assume that the MPU is the syllable (or word). Kawamoto et al. (1998) argued that purely parallel models could account for the regularity by position of regularity interaction if plosivity of the initial segment is taken into account and if the segment is the MPU. As illustrated in Figure 1, when the irregular grapheme is at position 1, targets beginning with plosives as well as non-plosives manifest the regularity effect because phonation cannot begin until the initial segment reaches threshold. When the irregular grapheme is at position 2, the 2nd segment takes longer to reach threshold for irregular graphemes than for regular graphemes. However, acoustic latency is longer only for irregular targets beginning with plosives; no effect of regularity is predicted for targets beginning with non-plosives. This interaction of plosivity and regularity for targets with the irregular grapheme at position 2 has been found (Cortese, 1998; Kawamoto et al., 1998). Finally, when the irregular grapheme is at position 3, only targets beginning with an /s/ followed by a plosive might manifest an effect, but only if a voice-key is used. On this account, the regularity effect diminishes from left to right because the proportion of stimuli that manifest an effect that can be detected acoustically diminishes from left to right. Roberts et al. (2003) rejected the account proposed by Kawamoto et al. (1998) because coarticulation was argued to be ubiquitous and thus the segment could not be the MPU. However, the coarticulation argument has been rebutted (see above). Moreover, Roberts and colleagues never provided an account of the regularity by plosivity interaction.

Cortese (1998) also reported simulations based on serial and parallel models showing that targets beginning with plosive as well as non-plosive targets predicted a regularity effect at position 2, but not the interaction. We argue that models fail to predict the plosivity by regularity interaction at position 2 because the naming latency predictions assume that the MPU is the syllable (or word) and that the dependent measure is acoustic latency. If the MPU is the segment, sequential and parallel models should account for the plosivity by regularity interaction at position 2. Thus, the crucial distinction is not whether processing is serial or parallel, but whether the MPU is the segment or the syllable.

## Final remarks

The segment MPU suggests that written word processing can be highly incremental, with the degree of incrementality varying across individuals and with stimulus and task demands. More importantly, articulatory, and acoustic effects implied by the segment MPU also affect assumptions about earlier encoding stages.

### Conflict of interest statement

The authors declare that the research was conducted in the absence of any commercial or financial relationships that could be construed as a potential conflict of interest.

## Footnotes

^1^Dell et al. (1993) use a simple recurrent network to produce segments sequentially without a stage that encodes segments to slots in a phonological frame.

^2^Some models (e.g., Dell et al., 1993) have been implemented as simple recurrent connectionist networks that generate each segment (or its features) after a fixed number of processing cycles without any buffering.

^3^If the effect is localized to the initial segment, measuring the duration of the entire word would miss the effect for targets beginning with plosives.
